# A case report of typhlitis during novel use of ropidoxuridine–capecitabine–radiotherapy for treatment-naïve rectal cancer

**DOI:** 10.1007/s00280-023-04561-4

**Published:** 2023-06-27

**Authors:** Charles A. Kunos, Richard Piekarz, Jerry M. Collins, Timothy J. Kinsella

**Affiliations:** 1 Department of Radiation Medicine, Markey Cancer Center, University of Kentucky, 800 Rose Street, C111, Lexington, KY 40536-0293 USA; 2 Cancer Therapy Evaluation Program, National Cancer Institute, Rockville, MD USA; 3 Developmental Therapeutics Program, National Cancer Institute, Rockville, MD USA; 4 Warren Alpert Medical School, Brown University, Providence, RI USA

**Keywords:** Ropidoxuridine, Capecitabine, Radiotherapy, Rectal cancer, Typhlitis

## Abstract

**Background:**

Rectal carcinomas are tumors that arise from the last 12 cm of the large intestine closest to the anus. They generally have a modest prognosis exacerbated by a high local recurrence rate if radiosensitizing chemotherapy is not given during radiotherapy. This case report discusses the clinical trial treatment of a patient with rectal adenocarcinoma by a new ropidoxuridine–capecitabine–radiotherapy combination. This case report is novel due to the patient’s participation in an accelerated titration phase I clinical trial and the resultant rare adverse event of treatment-related sigmoid typhlitis.

**Case presentation:**

The patient was an 82-year-old female who noticed hematochezia and change in stool caliber over a period of 3 months. A rectal mass was identified by biopsy as a microsatellite stable adenocarcinoma. A planned total neoadjuvant treatment involved eight cycles of leucovorin calcium (folinic acid)–fluorouracil–oxaliplatin (mFOLFOX6) chemotherapy, followed by a clinical trial combination of ropidoxuridine–capecitabine–radiotherapy, prior to definitive surgery. The patient began daily intensity modulated pelvic radiotherapy with concurrent twice-daily oral ropidoxuridine and twice-daily oral capecitabine to be given over 6 weeks. After 14 days of ropidoxuridine–capecitabine–radiotherapy, the patient developed sigmoid typhlitis requiring a 10-day hospitalization and 14-day disruption of treatment. The patient died 27 days after the start of ropidoxuridine–capecitabine–radiotherapy. This adverse event was listed as a definite attribution to the ropidoxuridine–capecitabine treatment; pharmacokinetic and pharmacodynamic data showed low ropidoxuridine metabolite DNA incorporation and high capecitabine metabolite concentration. The accelerated titration phase I clinical trial has been subsequently closed to accrual (NCT04406857).

**Conclusions:**

We believe this case report demonstrates the decision-making process for terminating a phase I accelerated titration designed clinical trial. The report also presents the rare complication of sigmoid typhlitis as a treatment-attributed adverse event. In this case, a ropidoxuridine–capecitabine combination was used as an investigational radiosensitizing treatment now with a narrower future clinical development pathway.

## Background

Typhlitis is a neutropenic enterocolitis of bowel characterized by medically significant but not immediately life-threatening fever, abdominal pain, and change in bowel habit with ileus [1]. Its pathogenesis remains multifactorial, with mucosal injury from infiltrative tumor, cytotoxic radiotherapy or drug effect, profound neutropenia, and impaired immunity against translocated bacterial pathogens all acting as causative factors. Diagnostic abdominopelvic ultrasound (US) or computed tomography (CT) confirms the diagnosis when the bowel wall is found to be thick (> 4 mm, transverse) over a protracted length (> 30 mm, longitudinal). Mortality risk is high due to consequential bowel ischemia, necrosis, hemorrhage, perforation, and multisystem organ failure. An estimated 5% of neutropenic cancer patients develop this condition [2].

New primary colorectal cancers are diagnosed in an estimated 151,030 men and women in the United States, and an estimated 53,580 patients are projected to die of colorectal-related disease in 2022 [3]. For tumors arising in the rectum, total mesorectal excision (TME) with pararectal lymphadenectomy is the main treatment modality. Total neoadjuvant treatment (TNT) precedes surgery for advanced-stage (T3–4 or N +) disease, with adjuvant chemotherapy having an established albeit controversial role after surgery [4–6]. In TNT schemes, radiotherapy most often involves daily pelvic radiation concurrent with one or more radiosensitizing chemotherapies [7, 8]. However, this treatment remains unsatisfactory, with 79% of surgical specimens demonstrating persistent disease [9]. For context, a 4% rate of typhlitis occurs after daily pelvic radiation concurrent with capecitabine [9]. As such, research activity focuses on early phase trials of novel radiosensitizing chemotherapy–radiotherapy combinations.

The phase I investigation offers a preliminary description of adverse events associated with chemotherapy–radiotherapy administration typically in a chemotherapy drug dose-dependent manner. The effectiveness of phase I evaluations depends upon patient cohort size, the rapidity of dose escalation, and the number of contributing treatment centers (10). A phase I cohort size of three to six patients affords a more accurate evaluation of toxicity and lessens the chance that serious adverse events are not recognized. Conversely, an accelerated titration phase I investigation might recruit single patient cohorts for rapid intrapatient or interpatient drug dose escalation [10]. To reduce the chance that serious adverse events are not missed in the accelerated design, observation of a lower grade of adverse event (i.e., grade 2 rather than the more typical grade 3) at a particular dose level ceases accelerated titration and prompts cohort expansion to an additional three to six patients [10]. This case report discusses the accelerated titration phase I investigation treatment of a single patient with rectal adenocarcinoma by a new ropidoxuridine–capecitabine–radiotherapy combination and the resultant life-threatening sigmoid typhlitis as well as associated pharmacokinetic and pharmacodynamic data.

### Case presentation

The patient presented as an 82-year-old woman who noticed hematochezia and change in stool caliber over a period of 3 months. At initial presentation, there were no constitutional symptoms including weight changes, fevers, chills, myalgias, or anorexia. Her social history was remarkable for 40-pack year smoking habit. Her past medical history was significant for depression, chronic obstructive pulmonary disease (emphysema), coronary artery disease complicated by hypercholesterolemia, and essential hypertension. Her family history was significant for breast, ovarian, lung, and bladder cancers; there was no personal or family history of colorectal cancer.

On initial examination, she had a 5 cm palpable rectal mass 8 cm from the anal verge, which was also visible as a 5.5 cm heterogenous soft tissue mass on contrasted abdominopelvic magnetic resonance imaging (MRI). The patient had normal appearance of genitourinary organs and the remainder of the examination was unremarkable. Contrasted CT imaging of the chest and liver were negative for metastatic lesions; pararectal lymphadenopathy was confirmed on contrasted MRI of the pelvis. She had a carcinoembryonic antigen (CEA) level of 8.2 ng mL^−1^. Given her initial diagnostic imaging, a recommendation was made for diagnostic colonoscopy and core needle biopsy for pathologic tissue evaluation and mismatch repair genotyping. Histopathology revealed a moderate-grade adenocarcinoma microsatellite stable with a clinical stage of stage IIIB, cT4a, cN1b, cM0.

Because of her untreated nonmetastatic disease, Eastern Cooperative Oncology Group performance status of 0, and adequate organ function, she was recruited to the National Cancer Institute (NCI) Experimental Therapeutics Clinical Trial Network (ETCTN) protocol #10,410, an accelerated-titration phase I study testing the addition of radiosensitizing oral ropidoxuridine (IPdR) to the usual concurrent capecitabine–radiotherapy combination for rectal cancer (NCT04406857). The accelerated titration design (Table 1) was based on prior phase I trial ropidoxuridine–radiotherapy experience [11]. NCI Central Institutional Review Board (CIRB, Bethesda, Maryland) granted approval for conduct of the trial; the patient provided written informed consent. Her first pretrial treatment step in the TNT schema was eight cycles of leucovorin calcium (folinic acid, 400 mg m^−2^ day 1), fluorouracil (400 mg m^−2^ day 1 and then continuously 2400 mg m^−2^ day^−1^ on days 1 and 2), plus oxaliplatin (85 mg m^−2^ day 1) chemotherapy (mFOLFOX6) repeated every 2 weeks starting in November 2020. Her second on trial step of TNT involved daily intensity modulated radiation therapy (IMRT) for 5 days per week to a dose of 50.4 Gy in 28 fractions of 1.8 Gy using 6 megavolt photons beginning in March 2021. Concurrent oral twice-daily ropidoxuridine 75 mg m^−2^ for 7 days per week and oral twice-daily capecitabine 875 mg m^−2^ for 5 days per week began on day 1 of radiotherapy. The investigational ropidoxuridine–capecitabine combination was to finish on day 28 of radiotherapy. Plasma ropidoxuridine (IPdR) pharmacokinetic assay and percent iododeoxyuridine (IUdR, a short-lived metabolite of IPdR) incorporation into granulocyte DNA pharmacodynamic assay were collected on day 8.

**Table 1:** Phase I accelerated titration design of protocol #10,410 of 5-iodo-2-pyrimidinone-2’-deoxyribose (IPdR, ropidoxuridine)^1^

Part A level^2^	Radiation	Capecitabine	Ropidoxuridine	Part B level^2^	Radiation	Capecitabine	Ropidoxuridine
1A	50.4 Gy	825 mg m^−2^ BID	75 mg BID	1B	50.4 Gy	825 mg m^−2^ BID	75 mg QD
2A	50.4 Gy	825 mg m^−2^ BID	150 mg BID (+ 100%)	2B	50.4 Gy	825 mg m^−2^ BID	150 mg QD (+ 100%)
3A	50.4 Gy	825 mg m^−2^ BID	300 mg BID (+ 100%)	3B	50.4 Gy	825 mg m^−2^ BID	300 mg QD (+ 100%)
4A	50.4 Gy	825 mg m^−2^ BID	600 mg BID (+ 100%)	4B	50.4 Gy	825 mg m^−2^ BID	600 mg QD (+ 100%)
5A	50.4 Gy	825 mg m^−2^ BID	1200 mg BID (+ 100%)	5B	50.4 Gy	825 mg m^−2^ BID	1200 mg QD (+ 100%)
				6B	50.4 Gy	825 mg m^−2^ BID	1800 mg QD (+ 50%)
				7B	50.4 Gy	825 mg m^−2^ BID	2400 mg QD (+ 33%)

*BID* twice daily, *Gy*  Gray, *QD* once daily. Parentheses indicate intensity of dose escalation

^1^Radiation and capecitabine are given Monday to Friday. On the days of radiation, ropidoxuridine is taken on an empty stomach within 30 min to 2 h before radiation (part A and B) and then again in the evening (part Aonly)

^2^The starting dose level of daily ropidoxuridine 75 mg m^−2^ for 7 days per week was found to be safe in a phase I trial [11]. In part A, single patient cohorts are used until a treatment-related grade 2 nonhematologic toxicity or grade 3 neutropenia or thrombocytopenia (DLT1) is observed. Two additional patients are recruited at this dose level. If a second patient or more develops a treatment-related grade 2 nonhematologic toxicity or any of the three patients develops a treatment-related grade 3 nonhematologic or hematologic toxicity, then part A enrollment stops and part B expands at the once daily dose equivalent level (e.g., 75 mg BID = 150 mg QD). Otherwise, part A continues in single patient cohorts. In Part B, cohorts of three patients are used until a treatment-related grade 3 nonhematologic toxicity or grade 4 hematologic or gastrointestinal toxicity (DLT2) occurs. Three additional patients are entered at this dose level. If none experience DLT2, three patients enroll at the next dose level. If one of the next three patient experiences DLT2, then dose escalation stops and three patients are entered at the next lowest dose level if only three patients were treated at that dose level. If two or more patients experience DLT2, then dose escalation stops and three patients are entered at the next lowest dose level if only three patients were treated at that dose level. When one or less of six patients experience DLT2, this is considered the maximum tolerated dose and generally the recommended phase II dose [10]

She achieved a partial response to mFOLFOX6 without any unresolved grade 2 or higher adverse event before starting her ropidoxuridine–capecitabine–radiotherapy. After 14 days of ropidoxuridine–capecitabine–radiotherapy, the patient complained of a 3-day history of 6–10 stools per day. Laboratory investigation disclosed grade 2 neutropenia and grade 3 thrombocytopenia. All trial therapies were stopped. Three days later, she was evaluated for grade 3 abdominal pain, grade 4 neutropenia, and grade 4 diarrhea. She was hospitalized for grade 4 sigmoid typhlitis when discovered on same-day abdominopelvic CT. Intravenous hydration and antibiotics were started. At 10 days after hospital admission, she progressed to pneumoperitoneum and bacterial sepsis (*Streptococcus bovis*) despite aggressive supportive care and intravenous antibiotics. She died from typhlitis-related bacteremia and multiple organ failure 27 days after the start of ropidoxuridine–capecitabine–radiotherapy. This grade 5 adverse event was listed as a definite attribution to the ropidoxuridine–capecitabine treatment. This event suspended the phase I accelerated titration portion of the trial; the trial closed to all accrual in December 2022.

A sensitive high-pressure liquid chromatography assay for percent IUdR incorporation into granulocyte DNA [11] found no incorporation of this metabolite into her granulocytes on day 8. Pharmacokinetic analyses [11] detected no IPdR plasma levels over 4 h of sampling; IUdR plasma levels peaked at 0.070 micromolar 30 min following the morning 75 mg ropidoxuridine dose and fell to less than 0.020 micromolar 4 h after the dose. Her pharmacokinetic analyses for capecitabine and its metabolites revealed a peak fluorouracil concentration of 1.2 micromolar at 2 h of the morning 825 mg m^−2^ dose, which is 10 times higher than reported in a prior capecitabine pharmacokinetic study [12].

## Conclusions and discussion

This case report describes a novel radiosensitizing strategy of ropidoxuridine–capecitabine during radiotherapy for TNT management of rectal carcinoma and treatment-related sigmoid typhlitis. Persistent disease at surgery after TNT therapies associate with a high recurrence rate and, therefore, inspires the development of novel radiosensitizing chemotherapy combinations in early phase clinical trials. In the example of ropidoxuridine–capecitabine during radiotherapy, there may have been beneficial tumor response but injury to other portions of the bowel, i.e., the sigmoid colon in this case, where neutropenic enterocolitis developed. Despite the daily ropidoxuridine 75 mg m^−2^ for 7 days per week being found safe in a first-in-human phase I trial [11], the ropidoxuridine–capecitabine combination was associated with toxicity in this case. While causality is difficult to ascertain, the timing and sequence of neutropenic enterocolitis argue that the medical events in this case are related to the intended intervention for the patient’s rectal carcinoma. One interpretation of the day 8 pharmacokinetic and pharmacodynamic data is that the ropidoxuridine and its metabolites did not contribute to the observed sigmoid typhlitis in the patient as there were diminished levels of these compounds in the blood. The undetectable level of percent IUdR incorporation into granulocyte DNA of the patient supports this notion. Conversely, the day 8 pharmacokinetics of capecitabine and its metabolite levels indicated high (up to 10x) fluorouracil concentration in the patient, more so than expected [12]. It is easy to speculate that such high levels of fluorouracil might indicate that the patient was heterozygous for the dihydropyrimidine dehydrogenase phenotype, which would raise fluorouracil blood levels, although no marked adverse events were noted during the eight cycles of leucovorin calcium (folinic acid)–fluorouracil–oxaliplatin therapy. A mutual kinetic interaction between fluorouracil and ropidoxuridine was not anticipated based on canine preclinical models [13, 14]. Ropidoxuridine cellular metabolism depends upon the deoxythymidine salvage pathway [14], and complex mutual kinetic interactions between thymidine kinase and thymidylate synthase or thymidine phosphorylase might also account for an observed high level of fluorouracil.

Second, the ropidoxuridine–capecitabine combination was considered investigational and studied on clinical trial, whereby informed consent for treatment was obtained from the patient. Aspects to consider in utilizing an accelerated titration design involve rapid initial dose escalation and intrapatient or interpatient variation in observable toxicity [10]. Where this trial unravels is in the single case accrued to the ropidoxuridine–capecitabine–radiotherapy treatment which obscures any ability to analyze its intended dose-toxicity model. Out of this report, we suggest that the investigators utilizing an accelerated titration design involving rapid initial dose escalation pay close attention to lower grade adverse events (i.e., grade 2 rather than the more typical grade 3) as an early signal of toxicity and adhere to protocol-specific dose-escalation rules for investigational agent dose titration. Given that advanced-stage rectal carcinomas associate with a poor prognosis in at least partly due to a high local recurrence rate, novel treatment strategies studied in early phase clinical trials remain desirable. This case report describes a relatively rare toxicity of sigmoid typhlitis definitely related to radiosensitizing chemotherapy during radiotherapy. Further research of dual-agent radiosensitizing chemotherapy during radiotherapy in accelerated titration phase I trials should be performed as this case report is limited to a single patient.

## Data Availability

Data sharing is not applicable to this article as no data sets were generated or analyzed during the current study.

## Abbreviations

Gy: Grays
IPdR: Ropidoxuridine
mm: Millimeters

## References

[1] United States Department of Health and Human Services (2017) Common Terminology Criteria for Adverse Events (CTCAE), version 5.0. Bethesda, MD: DHHS, 5: 43.

[2] Gorschlüter M, Mey U, Strehl J, Ziske C, Schepke M, Schmidt-Wolf IG, Sauerbruch T, Glasmacher A (2005). Neutropenic enterocolitis in adults: systematic analysis of evidence quality. Eur J Haematol.

[3] American Cancer Society (2020). Colorectal cancer facts & figures 2020–2022.

[4] Bosset JF, Calais G, Mineur L (2014). Fluorouracil-based adjuvant chemotherapy after preoperative chemoradiotherapy in rectal cancer: long-term results of the EORTC 22921 randomised study. Lancet Oncol.

[5] Maas M, Nelemans PJ, Valentini V (2015). Adjuvant chemotherapy in rectal cancer: defining subgroups who may benefit after neoadjuvant chemoradiation and resection: a pooled analysis of 3,313 patients. Int J Cancer.

[6] Kulaylat AS, Hollenbeak CS, Stewart DB (2016). Adjuvant chemotherapy improves overall survival of rectal cancer patients treated with neoadjuvant chemoradiotherapy regardless of pathologic nodal status. Ann Surg Oncol.

[7] Allegra CJ, Yothers G, O'Connell MJ, Beart RW, Wozniak TF, Pitot HC, Shields AF, Landry JC, Ryan DP, Arora A, Evans LS, Bahary N, Soori G, Eakle JF, Robertson JM, Moore DF, Mullane MR, Marchello BT, Ward PJ, Sharif S, Roh MS, Wolmark N (2015). Neoadjuvant 5-FU or capecitabine plus radiation with or without oxaliplatin in rectal cancer patients: A phase III randomized clinical trial. J Natl Cancer Inst.

[8] Van Cutsem E, Twelves C, Cassidy J (2001). Oral capecitabine compared with intravenous fluorouracil plus leucovorin in patients with metastatic colorectal cancer: results of a large phase III study. J Clin Oncol.

[9] O'Connell MJ, Colangelo LH, Beart RW, Petrelli NJ, Allegra CJ, Sharif S, Pitot HC (2014). Capecitabine and oxaliplatin in the preoperative multimodality treatment of rectal cancer: surgical end points from National Surgical Adjuvant Breast and Bowel Project trial R-04. J Clin Oncol.

[10] Ivy SP, Siu LL, Garrett-Mayer E, Rubinstein L (2010). Approaches to phase 1 clinical trial design focused on safety, efficiency, and selected patient populations: a report from the clinical trial design task force of the national cancer institute investigational drug steering committee. Clin Cancer Res.

[11] Kinsella T, Safran H, Wiersma S, DiPetrillo T, Schumacher A, Rosati K, Vatkevich J, Anderson LW, Hill KD, Kunos C, Collins JM (2019). Phase I and pharmacology study of ropidoxuridine (IPdR) as prodrug for iododeoxyuridine-mediated tumor radiosensitization in advanced GI cancer undergoing radiation. Clin Cancer Res.

[12] Cassidy J, Twelves C, Cameron D, Steward W, O'Byrne K, Jodrell D, Banken L, Goggin T, Jones D, Roos B, Bush E, Weidekamm E, Reigner B (1999). Bioequivalence of two tablet formulations of capecitabine and exploration of age, gender, body surface area, and creatinine clearance as factors influencing systemic exposure in cancer patients. Cancer Chemother Pharmacol.

[13] Smith DE, Brenner DE, Knutsen CA, Deremer SJ, Terrio PA, Johnson NJ, Stetson PL, Ensminger WD (1993). Mutual kinetic interaction between 5-fluorouracil and bromodeoxyuridine or iododeoxyuridine in dogs. Drug Metab Dispos.

[14] Kinsella TJ, Schupp JE, Davis TW, Berry SE, Hwang HS, Warren K, Balis F, Barnett J, Sands H (2000). Preclinical study of the systemic toxicity and pharmacokinetics of 5-iodo-2-deoxypyrimidinone-2'-deoxyribose as a radiosensitizing prodrug in two, non-rodent animal species: implications for phase I study design. Clin Cancer Res.

